# Nanomaterials Research at a Primarily Undergraduate Institution: Transforming Nanorods, Undergraduate Research Communities, and Infrastructure

**DOI:** 10.1021/acsnanoscienceau.4c00005

**Published:** 2024-04-24

**Authors:** Katherine E. Plass, J. Kenneth Krebs, Jennifer L. Morford, Raymond E. Schaak, Joshua J. Stapleton, Adri C. T. van Duin

**Affiliations:** † Department of Chemistry, Franklin & Marshall College, Lancaster, Pennsylvania 17604, United States; ‡ Department of Physics, Franklin & Marshall College, Lancaster, Pennsylvania 17604, United States; § Department of Chemistry, Department of Chemical Engineering, Materials Research Institute, The Pennsylvania State University, University Park, Pennsylvania 16802, United States; ∥ Materials Research Institute, The Pennsylvania State University, University Park, Pennsylvania 16802, United States; ⊥ Department of Mechanical and Nuclear Engineering, Pennsylvania State University, University Park, Pennsylvania 16802, United States

**Keywords:** remote instrumentation, PUI, postsynthetic transformation, research community, undergraduate research, computational research, anion exchange

## Abstract

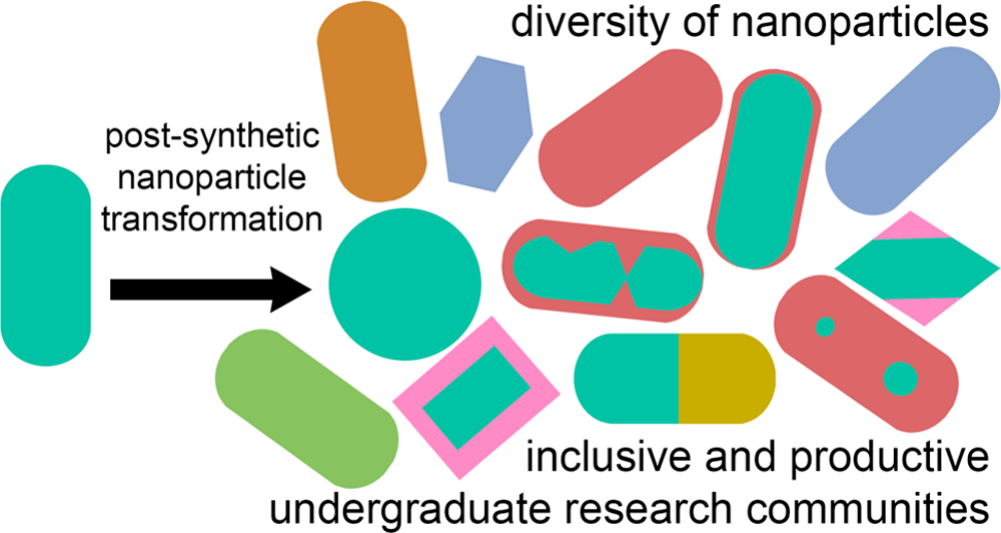

Undergraduate research transforms student’s conceptions of themselves as scientists and encourages participation and retention in science, technology, engineering, and mathematics (STEM) fields. Many barriers exist to carrying out scientifically impactful undergraduate research in nanomaterials at primarily undergraduate institutions (PUIs). Here, we share several practices and design principles that demonstrate pathways to overcome these barriers. Design of modular research projects with low entry barriers is essential. Postsynthetic transformation of nanoparticles is a field that enables such design and has been used successfully to advance nanoscience research while being achievable within undergraduate laboratories. Relatively large, inclusive research communities can be supported through the creation of opportunities with peer- and near-peer mentoring. We also share emerging strategies for enabling routine undergraduate access to transmission electron microscopy, which is one of the most mainstream characterization techniques in nanoscience yet is frequently absent from the infrastructure at undergraduate-focused institutions.

## 1 Introduction

Nanoscience is a multidisciplinary field that provides capabilities, insights, knowledge, and applications that focus on the unique behavior of matter at length scales that are intermediate between atoms and micron-scale systems. The continued strengthening and growth of this field rely, in part, on developing rational design strategies for constructing new nanomaterials and developing a diverse pipeline of well-prepared new scientists to tackle future challenges and opportunities.

Undergraduate research in nanomaterials provides students with a strong grounding in the theory and applications of nanoscience. In general, undergraduate research is known to be a powerful mechanism for encouraging student participation and retention in STEM fields, particularly for students from minoritized and marginalized groups.^1^ Positive undergraduate research experiences can transform a student’s identity and self-conception and improve their resilience and self-efficacy.^2,3^ The positive impacts of undergraduate research are amplified when students engage early in their college careers and these impacts continue to increase as long as students continue their undergraduate research experiences.^4^ Thus, it is important to involve as many students as possible in undergraduate research, especially early in their careers, and undergraduate research in the field of nanoscience can be particularly fruitful. Furthermore, it is important that this research be impactful and productive. Many barriers exist, however, to engaging undergraduates broadly in research and especially in nanoscience research.

Barriers to broad participation in undergraduate research can be even steeper at primarily undergraduate institutions (PUIs; institutions that award fewer than 20 Ph.D. degrees each year). Overcoming these barriers is particularly important when PUIs and other small colleges are the baccalaureate origin of a substantial percentage of STEM PhDs in the US (9% from 2010 to 2020).^5^ Small colleges also have an outsized effect on the pipeline of women and students from under-represented groups.^6^ The Primarily Undergraduate Nanomaterials Cooperative (PUNC) recently articulated the numerous challenges that face nanoscience researchers at PUIs.^7^ These challenges include heavy teaching loads, small departments where nanomaterials researchers may be isolated, limited facilities, and a lack of graduate students.

This Perspective focuses on approaches we have uncovered in our nanoscience research and education efforts that enable broad participation of numerous undergraduate students in impactful nanomaterials research at a PUI. The specific approaches we describe have been developed in the Plass laboratory at Franklin & Marshall (F&M) College over the past 15 years, where undergraduate research has included developing phase-selective syntheses of copper chalcogenides,^8−11^ investigating their plasmonic behavior,^11,12^ and establishing new methods for postsynthetic transformation (PST).^13−16^ These efforts have been enhanced by collaborators at F&M and Penn State University (PSU, an R1 institution, meaning it grants doctoral degrees and has a very high research productivity), who helped to explore new ventures to benefit students. F&M is a small, relatively research-intensive, liberal arts college with only 2200 students (for comparison, PSU’s main campus has ∼40,000 students). F&M students and faculty have close interactions in small classes (25–30 students). F&M’s strong history of publication and grant-seeking has helped ensure a research infrastructure with essential laboratory facilities for nanoparticle synthesis, transformation, purification, and routine characterization. It has also led to F&M being one of the top 5 PUI origins of chemistry Ph.Ds.^5^ While we highlight specific approaches developed in the Plass laboratory, we propose that they represent examples of a broader design principle for productive and inclusive undergraduate-led research: **to give students agency within a modular and scaffolded research structure, a strong peer-mentoring community, and validation through a low entry-barrier to high-impact projects and near-peer external collaboration.** In this Perspective, we first describe how postsynthetic transformation of nanoparticles has afforded a research structure in which to design impactful projects amenable to the inclusion and integration of undergraduate students with only introductory chemistry experience, which represents a low-barrier entry point in the undergraduate curriculum. We then focus on the various research communities that have been initiated and sustained to provide peer- and near-peer mentoring. The research team system is crucial for knowledge transfer by keeping a pipeline of students with different experience levels, allowing involvement of students early in their college careers, including all interested students, and enabling flexibility to support a variety of student needs. This experimental work has been supported by a strong near-peer network of graduate students in the Schaak laboratory at Penn State, which is fostered by both virtual and in-person interactions. A blossoming computational research community called the “nanobots” research project was also developed in collaboration with the van Duin group at Penn State. This collaboration was driven by the collective efforts of F&M students, along with F&M faculty Krebs (physics) and Morford (chemistry). Lastly, we describe how we have enabled routine PUI access to transmission electron microscopy (TEM), an indispensable characterization technique in nanoscience research. An innovative approach to remote instrument access with Penn State’s Materials Characterization Laboratory (MCL) was developed and has transformed undergraduate research productivity, as it allows F&M students to use scanning transmission electron microscopy (STEM) imaging with energy dispersive X-ray spectroscopy (EDS) element mapping to evaluate the nanoparticle samples that they make in the laboratory.

## 2 Transforming Nanomaterials—Selecting High-Impact Research Areas with Low Entry-Barrier and Modularity

Undergraduate research, especially at PUIs, is carried out in short sessions by students and faculty, for whom a majority of their time is devoted to classes. There is limited time to carry out training and the implementation of projects. This reality makes it essential to design undergraduate research projects that maximize the likelihood of achieving publishable data and allow for the work of several students to be knit together into a final publication. The study of PSTs of nanoparticles allowed us to construct projects in which students can quickly learn specific tools and then collaborate with other students to apply them. By doing so, students gain knowledge about how to design new nanomaterials. Identification of new PSTs and their cooperativity is a powerful route toward realizing an incredible diversity of particles. It is also amenable to the modularity and low entry-barrier required to achieve the most impactful and productive undergraduate research experiences.

It is well-known within the nanoscience community that a broad scope of nanomaterials can be directly synthesized, but despite decades of research, it still remains challenging to design complicated nanomaterials with precisely controlled sizes, shapes, phases, and compositions, beyond a few well-studied systems. It can be even more challenging to design nonspherical multicomponent nanoparticles with specific placement of different chemical species, as can be required for advanced catalytic, optical, thermoelectric, magnetic, and active matter applications. Synthetic routes to multicomponent nanoparticles, which incorporate multiple materials into a single nanoparticle, include seeded growth and directed deposition.^20−24^ Postsynthetic transformation of nanoparticles is an alternative route whereby synthon particles are first synthesized using direct methods and then in subsequent steps the sizes, shapes, compositions, and/or phases of the synthon particles are altered.^25−28^ Copper sulfide nanoparticles have proven to be particularly amenable to PST with an increasingly large toolbox of PST reactions including cation exchange,^26−32^ anion exchange,^14−16,26,33^ shape change,^17,34,35^ and redox doping.^18,30,36,37^

One benefit of PST is that it leverages and builds upon robust and well-established syntheses of existing nanomaterials. Cu-deficient copper chalcogenide nanomaterials have found widespread use as facile starting materials for various forms of PST due to their tolerance of large vacancy concentrations that help to facilitate such transformations, creating an array of single- and multiple-component metal chalcogenide nanoparticles.^38^ Undergraduate research students who are inexperienced can quickly learn to synthesize various hexagonally close-packed phases of copper sulfide nanospheres, nanoplatelets, and nanorods.^39^ We find that the nanorod synthesis can be the most challenging, with new students tending to make nanospheres instead of nanorods during their first few attempts. This dependence on robust syntheses as a starting point for a research project is important in the undergraduate research context. Having to extensively troubleshoot a reaction that is not consistent is time-consuming for all researchers, but when the start-to-finish time scale of undergraduate research projects is measured in units of months, such setbacks can ruin productivity.

**Figure 1:**
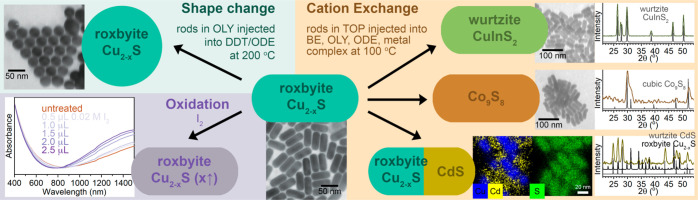
Toolbox of robust, low entry-barrier PSTs of roxbyite nanorods adapted from the literature and implemented by undergraduate researchers. (Top left) Shape change with 1-dodecanethiol (DDT) solvent exposure with TEM showing the transition to spheres.^16,17^ Adapted from ref (16). Copyright 2023 American Chemical Society. (Bottom left). Oxidation with I_2_ with UV/visible/NIR absorption spectra showing the blue-shift in LSPR absorption with increased I_2_ exposure.^13,18^ Adapted from ref (13). Copyright 2020 American Chemical Society. (Right) Partial and complete cation exchanges by injection of rods in trioctylphosphine (TOP) into a solution of dibenzyl ether (BE), oleylamine (OLY), and 1-octadecene (ODE). Examples of full conversion to Co_9_S_8_ and CuInS_2_ occurred with the reported phase and retention of the nanorod morphology. Partial exchange with CdS is shown with PXRD including both roxbyite Cu_2–*x*_S and wurtzite CdS and STEM-EDS mapping showing partial conversion to CdS with the expected regioselective incorporation from the tip of the rods.^13,19^

After students successfully synthesize copper sulfide nanoparticle synthons, they can apply a modular suite of PSTs to generate a diverse array of derivative products. Cation exchange is the exemplar PST, widely used to create nanoparticle phases and morphologies that are difficult to directly synthesize.^40−42^ Complete cation exchange reactions replace all of the copper cations in the copper sulfide nanoparticles, thereby completely changing the metal in the metal sulfide. Partial cation exchange reactions can create highly elaborate, multicomponent nanoheterostructures that have different materials in different regions, due to the regioselective nature of such reactions.^39,43−48^Figure 1 shows the toolbox of published PSTs that we have found to have the low entry-barrier and robust outcomes required for use in PUI research. Partial cation exchange, as implemented in a detailed Methods paper,^19^ are generally robust and have been used sequentially to amplify structural complexity.^39,44,49^ Stoichiometrically limited partial cation exchange has proven to be particularly useful. To use this approach, students make a solution of a dissolved metal complex or salt that can be stored for several weeks if it is purged with Ar and kept in a desiccator. The Cd^2+^, Co^2+^, and In^3+^ exchanges are easily implemented while Zn^2+^ exchange requires more care. (Generally, the terminology “*M*^*n*^^+^ exchanges” refers to the incoming cations from solution that enter the nanocrystal concomitant with expulsion of Cu^+^ cations from the copper sulfide nanoparticles into solution during the PST reaction.) Students generally observe retention of shape and phase, except for the reported shape- and size-dependent phase conversion upon Co^2+^ exchange;^50,51^ these results are expected based on literature. In addition to complete and partial cation exchange, two other PST reactions have been adapted from the literature and proven to be amenable to implementation in undergraduate research. In the first reaction, the plasticity of the roxbyite nanorods makes their shapes controllably deformable by incubation in alkylthiol solvents.^16,17^ In the second reaction, the copper vacancy concentration can be altered by oxygen exposure^11,52−54^ and addition or removal of Cu^+^ ions.^18,30,36,37^ The doping level then alters the NIR LSPR frequency.^12,18,54,55^ We have used surface passivation to stabilize the Cu^+^-vacancy concentration.^12^ The Cu^+^-vacancy concentration can be increased by postsynthetic treatment with I_2_.^13,17,18^ These PSTs can also be used cooperatively. Increasing the copper deficiency with I_2_ facilitates solid-state ion diffusion, which is a crucial step in PSTs. This accelerates cation exchange^13^ (Figure 1) and facilitates shape change.^17^

**Figure 2:**
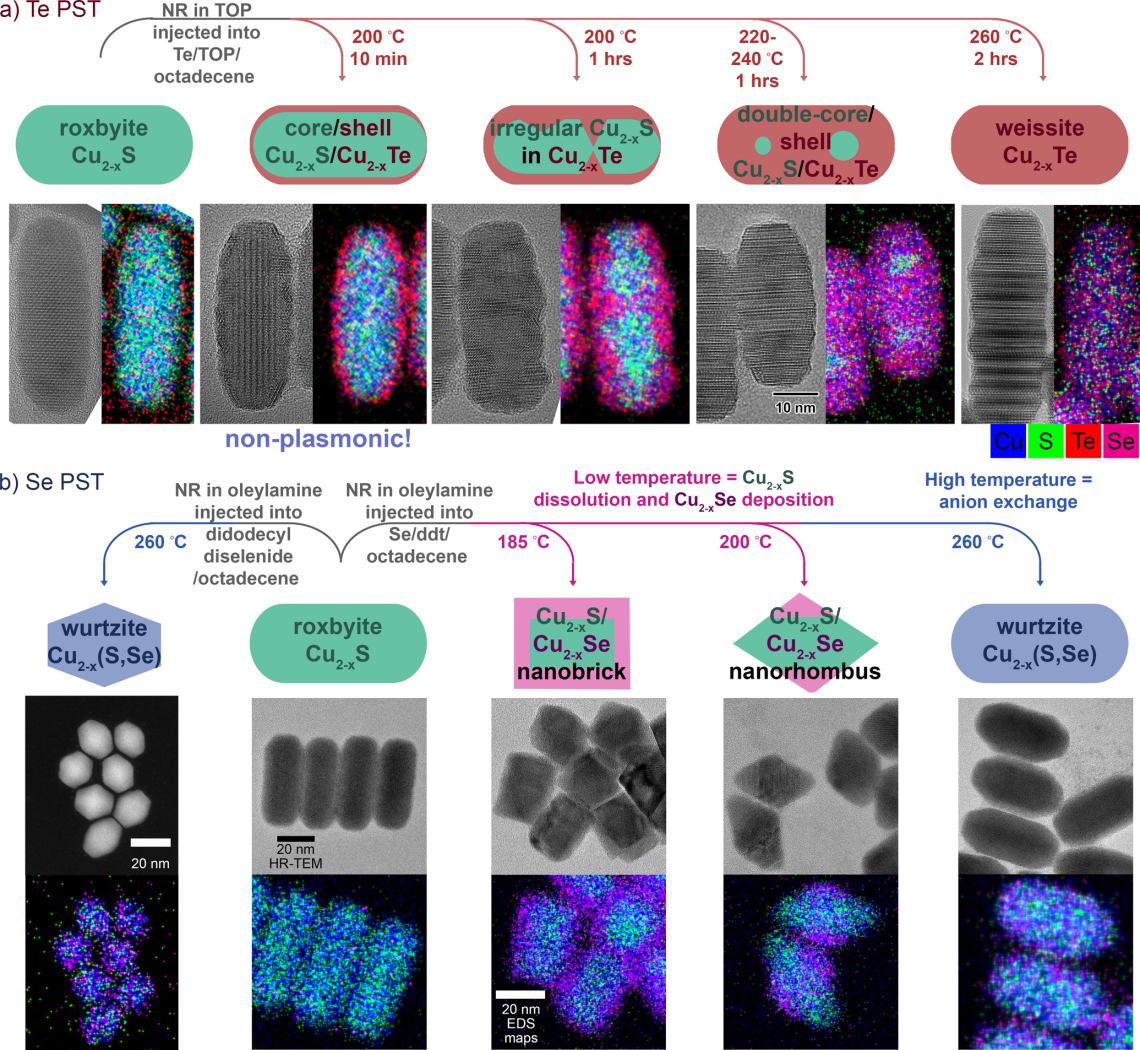
Toolbox of PSTs developed by undergraduates to alter the anionic component of Cu_2–*x*_S nanorods. (a) Te^2–^ anion exchange proceeds through stages of different Cu_2–*x*_S/Cu_2–*x*_Te heterostructure regioselectivities before complete exchange.^14^ The plasmon resonance changed with transformation as indicated.^15^ Adapted from ref (14). Copyright 2021 American Chemical Society. (b) Alteration with Se^2–^ is more complicated, with changes in shape, phase, and regioselectivity with reaction temperature due to different mechanisms. The transformation with Se^2–^ is simplified by use of a dialkyldiselenide reagent for anion exchange.^16^ Adapted from ref (16). Copyright 2023 American Chemical Society.

**Figure 3:**
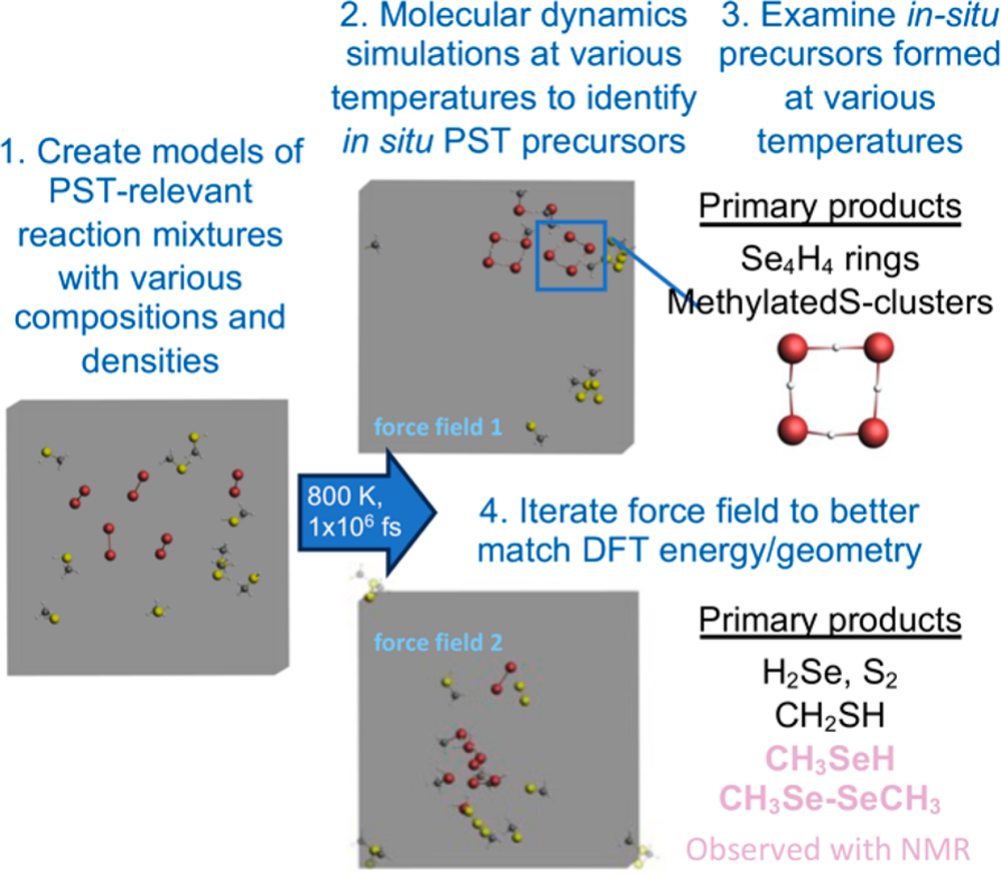
Example of the computational modeling process in which Nanobots Research Project students are engaging. Step 1. Students create a model containing Cu, S, Se, C, and/or H inspired by the interactions between Cu_2–*x*_S nanorods, dodecanethiol, selenium, and octadecene that cause temperature-dependent PST. Step 2. Molecular dynamics simulations are run using ReaxFF. This generates various molecules that can be vetted against experiment data and DFT calculations in Step 3. Step 4. This data is shared iteratively with the van Duin group to improve the ReaxFF force field. Here is an example where the improved force field simulated alkyl selenide formation observed through NMR.^16^ Calculations and graphics used the Amsterdam Modeling Software.

**Figure 4:**
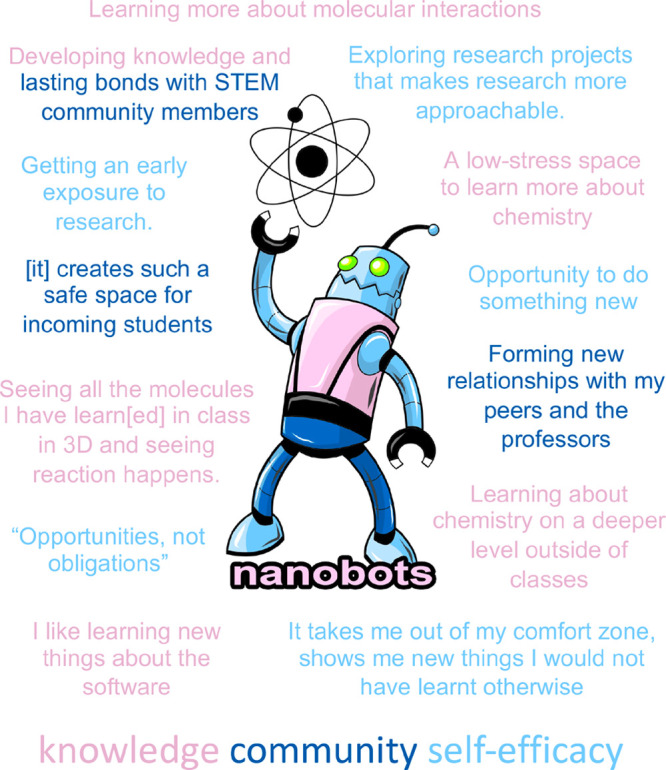
Student responses to the question, “What is your favorite thing about Nanobots?” with the Nanobots logo (created by Nick DelCore and used with permission). Responses have been color-coded into three rough categories. Pink entries emphasize knowledge acquisition; dark blue entries emphasize formation of a community; light blue entries emphasize an increased sense of being comfortable doing something new/doing research.

Inspired by the power of cation exchange to create such a diversity of multicomponent nanoparticles with straightforward application of robust PSTs,^38,39,44,49^ we recently focused on complementary methods to alter the anion component of roxbyite nanorods. Anion exchange or other anion-focused transformations are less studied than cation-focused transformations, but recent reviews highlight the rising interest in this topic.^26,28,56^ Cations are generally small relative to anions, and cations (particularly in copper chalcogenides) migrate rapidly throughout a crystal. Compared with cation exchange, anion exchange involves diffusion of much larger ions through the crystal lattice. This process can more easily destroy particles^57^ and commonly results in voids due to differences in the mobilities of incoming and outgoing ions; this results in the so-called Kirkendall effect.^25,58,59^ Despite this potential limitation, we adapted a published procedure^60^ to demonstrate Te^2–^ and Se^2–^ anion exchange in roxbyite copper sulfide nanorods with retention of the nanorod shape and size, and without Kirkendall void formation (Figure 2).^14,16^ Both exchanges retained the hexagonally close-packed anion sublattice to create metastable weissite Cu_2–*x*_Te and wurtzite Cu_2–*x*_Se phases. Alteration of the exchange conditions resulted in a variety of Cu_2–*x*_S–Cu_2–*x*_Te and Cu_2–*x*_S–Cu_2–*x*_Se nanoheterostructures. The extent of Te^2–^ exchange could be controlled by changing the time and temperature of the reaction. Low levels of exchange created a Cu_2–*x*_Te shell around a Cu_2–*x*_S rod. Defects then accelerated exchange along crystal planes perpendicular to the edge of the rods, resulting in irregular areas of Cu_2–*x*_Te amidst Cu_2–*x*_S. After phase segregation, Cu_2–*x*_S condensed into two small areas to give a Cu_2–*x*_S/Cu_2–*x*_Te double-core/shell structure (Figure 2a). Formation of a thin Cu_2–*x*_Te shell induced a phase change that altered the localized surface plasmon resonance of the particles.^15^ The greater similarity in the ion sizes of the S^2–^ and Se^2–^ anions (relative to the sizes of S^2–^ and Te^2–^) led to formation of solid solutions, instead of phase-segregated heterostructures, upon anion exchange. Changing the reaction time allowed for variation of the extent of Se incorporation. Varying the reaction temperature for the Se^2–^ exchange produced very different results than those observed for Te^2–^ exchange. Instead of changing the extent of exchange, the shape, phase, and composition were instead altered. Lower temperatures resulted in core–shell Cu_2–*x*_S/Cu_2–*x*_Se nanobrick or nanodiamond shaped particles (Figure 2b). The reaction mixture of Se, dodecanethiol, and octadecene formed different selenide compounds *in situ* at different temperatures, resulting in distinct mechanisms that led to distinct products. We first hypothesized that formation of dialkyl diselenide during the reaction was driving anion exchange at high temperatures and then validated this hypothesis through independently synthesizing this molecule and using it directly to form the same nanoparticle product (Figure 2b). This dependence of the outcome of the PST reaction on the *in situ* precursors and the experimental challenge of probing the reaction mixture directly inspired the computational modeling project, the nanobots research project, discussed below (Figures 3 and 4). The formation of these various copper chalcogenide nanoheterostructures could be useful to study the effects of nanostructures on photocatalysis, plasmonic photothermal therapy, Li-ion storage, thermoelectrics, and topological insulators, which are current applications of copper chalcogenide nanomaterials. Of particular interest to modular nanomaterials design, copper tellurides^62^ and selenides^40,48^ can be the starting materials for further cation exchange.

The ability to manipulate the anionic component of copper sulfides with PST begs the question of how these transformations intersect with existing PSTs that alter the vacancy concentration, shape, or cation component. How do the design rules guiding these individual transformations change when combined with a second transformation? Are design rules additive, or does one process change the course of the second? Combining cation exchange and metal deposition allowed us to uncover the design rules for cooperation of these PSTs.^63,64^ Multigeneration cation exchange suggests that new behaviors are likely to emerge.^44^ Regioselectivity is directed by similarities in crystal structure along different directions^39,65^ but the introduction of interfaces creates disordered areas where new cations are rapidly introduced.^44^ Selective cation exchange on Cu_2–*x*_Se/Cu_2–*x*_S dot-in-rod structures with Ag^+^ and Hg^2+^ shows that cation exchange proceeds selectively in the core to form the more thermodynamically favorable selenides.^48^ Would a similar selectivity convert Cu_2–*x*_Se/Cu_2–*x*_S nanodiamonds to Ag_2_Se/Cu_2–*x*_S? As we combine PSTs and compare the resultant regioselectivities with those observed when only one PST is carried out, we could consider a few “extreme case” outcomes. In the smallest perturbation, we might observe simple additivity; the design rules guiding the regioselectivity for each PST appear to be applied one after the other. In the greatest perturbation, consecutive PSTs may introduce sufficient structural instability such that particles are broken apart. Within those two extremes exist the opportunity for cooperativity of PSTs and the emergence of new design rules. This research question opens many avenues for exploration using a toolbox of robust copper sulfide nanoparticle syntheses and PSTs that undergraduate researchers can rapidly learn and then utilize creativity to explore unique ideas with potential highly impactful outcomes.

## 3 Transforming Research Communities—Flexible Opportunities and Mentoring to Create Supportive Criticality

The creation of new PSTs and understanding their cooperativity afford new opportunities to engage undergraduate research students. More opportunities overall allow us to engage students earlier and over longer times, as needed to fully realize the benefits of undergraduate research.^4^ To provide appropriate training while building belonging, students need to be part of an intellectually challenging and emotionally supportive community.^66^ They need to take ownership of a research project and build the skills and confidence to carry it out. They need to see the value in their work as authentic, impactful science that is of interest to the external scientific community. This requires interactions that ensure students that success comes only as a result of overcoming many challenges and routine failures, that they can start where they are, make mistakes, and continue to grow. It also requires interactions that model ever-deeper intellectual engagement and skill. We have developed three distinct research communities to inspire undergraduate researchers and normalize the uncertainty and errors that are inseparable from the research process. These augment internal research collaborations on oceanographic materials between Morford and Plass^67^ and ongoing collaborations^68^ and interactions through PUNC.

### Flexible, Team-Based Undergraduate-Led Research Uses Peer-Mentoring to Promote Productivity

In recent years, the number of students carrying out experiments to explore PST reactions in the Plass lab has grown to 8–11 students at any given time. The Plass lab has involved 65 undergraduates in experimental nanomaterials research over the last 16 years; 30 of those students have been involved since 2020. (Note that of these 30 students, only 2 chose non-STEM majors. With 18 students who are graduates or seniors, 40% are in graduate programs in chemistry or engineering.). This expansion has necessitated and led to a more inclusive research community. This larger group and the structures that have evolved to support it have created additional, more flexible opportunities for undergraduate research, enabled inclusion of more students earlier in their career, and allowed them to persist in their research.

Prior to 2020, it was typical to have ∼5 undergraduate researchers work for 10 weeks during the summer and ∼4 students work during the academic year, which amounts to 12 h/week (1 class). The disruption due to pandemic-related laboratory closures and online learning led students to feel isolated and concerned about getting more laboratory experience. As a result, we experimented with allowing shorter, more flexible experiences and including more students by creating research teams. Since 2020, 8–10 summer research students have worked for 5–10 weeks (paid primarily from grant funding with some additional college funds). The length of time is chosen in consultation with students and enables participation by students with heavier family obligations, for example. Research for academic year credit was also made more flexible. A research course, “Introduction to Problems in Chemistry,” has been introduced. This course enables first and second year students willing to commit 6 h per week to carry out research to receive half-course academic credit. This course enabled early career students with limited time availability to stay connected to their research community and retain their research skills. It quickly evolved, however. Upper-level students recognized this course as an opportunity to get involved in research, even when they did not have room in their academic plans for a full-credit research course. Others saw it as an opportunity to get research training ahead of a greater commitment, extending their period of research engagement. Overall, this increased flexibility and willingness to accept less-experienced students have resulted in a remarkably diverse and persistent research community. Now, 8–11 students signed up for research each semester. This diverse group spans a range of educational stages from prematriculation to postgraduation; 60% are female, 30% are international, 25% are students of color. The group has also included neurodivergent and LGBTQ+ students. The students were remarkably persistent in their work. For example, of the 8 students who participated in summer research in 2021, 7 continued to do further research during subsequent academic years (1–3 semesters), and 5 out of 6 eligible students returned in the summer of 2022. Students who choose not to do research in the Plass lab in subsequent summers do so because they have received competitive industrial internships or clinical experiences. Overall, students participate in an average of 2.5 semesters/summers of research.

Supporting so many students in the laboratory, including less-experienced students, requires careful construction of research teams for training and support. Each team consists of 3–4 students, combining those with both more and less experience. Each team works together to test a particular hypothesis regarding PSTs and to train on synthesis and 1–2 specific PSTs; a detailed project that was structured in this manner was described in Section I. In this model, more-experienced students train less-experienced students. Such peer mentoring provides leadership opportunities and builds confidence both in the mentor and in the mentee. Experienced students are challenged to articulate their understanding, gain confidence, correct misconceptions, or solidify knowledge in the process. Less experienced students learn in a “safe” environment where they can test ideas and make mistakes and know they will be understood by their peers. This peer-mentoring also gives new students the opportunity to “fail” in a safe way, which is essential for navigating research and promoting inspired and ambitious science.

Our recent publication, “Temperature-Dependent Selection of Reaction Pathways, Reactive Species, and Products during Postsynthetic Selenization of Copper Sulfide Nanoparticles,″^16^ demonstrates how a series of modular, low-entry barrier projects can be connected into a coherent story. In the summer of 2021, Brandon tested what happens when roxbyite rods were injected into a 1-dodecanethiol (DDT), selenium, 1-octadecene (ODE) solution at different temperatures, discovering an interesting transformation that only required two synthetic skill sets. Once this interesting system was identified, two students, Rebecca (Qi) and Valerie (Wanrui), worked together to understand the shape evolution at 185 and 200 °C throughout their 1-year senior research projects. Their work revealed the dissolution-deposition mechanism to make core/shell particles. In Spring 2022, Brandon resumed investigations into the anion exchange process that was observed at 260 °C. In Summer 2022, Brandon led a team with Mary (Chi Loi Thanh) and Eli. They undertook variations of the selenization procedure to try to understand the role of each component of the reaction mixture. Mary and Eli started using different thiols, which revealed the crucial role of DDT on the phase- and shape-evolution. This suggested that NMR evaluation of various combinations of the reactant at different temperatures would be helpful, and this was undertaken by Eli, Brandon, and Diya during the Summer and Fall of 2022. Finally, we hypothesized that dialkyl diselenides were actually causing the anion exchange, and a new student, Ronald (trained by Rebecca and Eli), stepped in during Spring 2023 to synthesize the molecule and demonstrate that it does cause anion exchange. Throughout this process, students consistently used the same scientific tools: nanorod synthesis, the selenization process that we developed, and characterization with PXRD, TEM, SEM-EDS, and STEM-EDS mapping. Note that careful scientific record keeping, including an electronic notebook system with strict naming conventions, detailed table-of-contents, and easily accessible data storage, is essential to final collation of data and attributing appropriate authorship. In addition to end-of-summer or semester reports, students frequently give data analysis presentations where they are pushed to synthesize and critically interpret their team’s findings.

Having such a large undergraduate laboratory at a PUI can appear controversial to some. It is a tenet of many undergraduate-focused research environments that close faculty-student interaction is one of the greatest strengths of PUIs and that faculty-student collaborative research exemplifies this. Indeed, these close faculty mentoring experiences have an important, lasting impact on students. Peer-mentoring does, in some ways, replace or supplement faculty interactions. When challenged about this potential drawback, students tend to argue that this large, team-based research structure augments their experience rather than detracts from it. They argue that a less faculty-driven structure gives them agency and that they are proud when they can show a new student how to use an instrument or explain a complicated concept. They often note that it can be much less intimidating to learn complicated concepts from other students than from faculty and that students work together to decide when and how to ask for help from faculty. They cited the sense of togetherness and community that came from working closely with other students on a project they all care about as being important to their sense of belonging.

### Flexible, Cooperation with R1 Laboratories for Near-Peer Mentoring and Community of Expertise

The graduate students in the Schaak laboratory at Penn State have formed a novel near-peer mentoring research community for the undergraduate research students in the Plass laboratory as well as a community of expertise for the PI (Plass). This interaction between a PUI and R1 laboratory is not a typical research collaboration with shared experimental plans and goals but is instead focused on sharing expertise and experiences. A few times each year, we hold a joint group meeting where undergraduate and graduate students give short presentations about their work. These meetings are relatively informal and are intended to allow detailed discussion of data and project goals. Plass also joins weekly Schaak lab group meetings through teleconferencing and hosts discussions about teaching, making the interactions synergistic and beneficial to everyone.

The near-peer research community formed by Schaak lab graduate students and F&M undergraduate students encourages resilience and models criticality of data and ideas while supporting people. These engagements between students from PUI and R1 institutions prepare F&M students for the depth and rigor of graduate studies in a way that is not typical of an exclusively PUI-based undergraduate research experience. The joint group meetings are held in-person at PSU or F&M. We intentionally start with lunch and time for informal discussion to help students get comfortable with one another. F&M students are briefed ahead of time to consider questions regarding their academics or career that they would like to ask a recent graduate. Topics of discussion include postgraduate career decisions and choosing a graduate school or mentor. Students also share failures, such as times they struggled in classes or made mistakes in lab. These shared experiences provide a crucial perspective on undergraduates’ current struggles. They normalize routine failure and inspire persistence. The data discussions model the depth of analysis expected at the graduate level, with many questions, shared ideas, and suggestions for experiments. These visits are deeply validating for undergraduate researchers. They find that they can speak the language of other scientists who are interested in their work. In the words of one undergraduate, they did not know they were doing “real research” until they saw how relevant it was to other people. These visits help to demystify graduate school, particularly for students unfamiliar with navigating the peculiar culture and opportunities it affords.

Frequent interaction of the Plass lab with the Schaak lab has been a powerful accelerator of research, allowing the undergraduate research team to more quickly overcome experimental obstacles by consulting with graduate students and discussing experimental design through virtual group meetings. As recently laid out by PUNC,^7^ a lack of graduate students is a major challenge to research at PUIs. Graduate students and postdocs are able to commit the time necessary to truly become experts in their field, working full time for years, while PUI faculty have heavy teaching loads that relegate research to a secondary concern. In our case, the graduate students at Penn State share their time, experience, and knowledge to discuss the PUI-led projects while themselves are engaging in important and unique professional development opportunities, including mentorship and outreach. Between direct laboratory experience, a detailed familiarity with recent literature, and a willingness to share unpublished findings, these discussions help to troubleshoot experimental challenges, shape the story, avoid or truncate unproductive pathways, and speed up the time to publication.

### The Nanobots Research Project—Including *Everybody* in Research to Understand PST Chemistry

The Nanobots Research Project represents an innovative strategy for engaging students in nanoscience research and building a research community. The project was named “nanobots” by the students to reflect the use of computers to model nanomaterials. This project is a convergence of several goals. Intrigued by the temperature-dependent role of *in situ* precursor formation in Se, dodecanethiol, and octadecene in altering the PST mechanism, as discussed in Section 2 (Figure 2b), we sought to more fully understand these reactions. We also sought to overcome the significant barriers to involvement in research by first- and second-year students. While a large and inclusive experimental group can “get students hooked” on research, the laboratory setting places practical limits on how many students can be involved. It also usually requires students to have completed their first year of college, a crucial time where they have to navigate the unspoken culture of college and STEM—the “hidden curriculum”. The success of the STEM Posse Program^69^ has demonstrated that marginalized students need a supportive mentor *from the beginning of their college career* and a cohort of other students experiencing and willing to share similar academic and nonacademic challenges. At F&M, Morford, Krebs, and Plass designed the Nanobots Research Program to be a space where we can form those mentoring relationships with students while providing them with the experiences that teach self-efficacy and help them feel a sense of belonging while they navigate the challenges of introductory STEM courses.

In collaboration with the van Duin at Penn State, the Nanobots program leverages the ReaxFF method for modeling reactive chemical systems including metal chalcogenide nanocrystals.^70−73^ The ReaxFF method provides opportunities to use a quantum-mechanics trained empirical force field to perform reactive molecular dynamics modeling of materials systems with many thousands of atoms.^70−73^ We have been training force fields on processes involving Cu, S, and Se as a basis for understanding the PST of copper sulfide to copper selenide. Our preliminary approach involves using molecular dynamics simulations to generate a variety of molecular species. We then extract various molecular species and compare their energies and optimized geometries between ReaxFF force fields and DFT methods (Figure 3).

The Nanobots Research Project has been carefully designed to allow a low entry-barrier and a rolling start. Students can join the group at any time in the semester. We hold weekly meetings led by upper-level students where we work together to do and interpret molecular dynamics calculations. Students use ReaxFF as implemented through the Amsterdam Modeling Suite either on their laptops or through F&M’s computational cluster. Introducing computational modeling through a graphical user-interface makes setting up calculations and visualizing results quite accessible to students. Students can start to contribute after a few hours of training, thanks to training videos developed in collaboration with the van Duin group. New students are welcomed throughout the academic year. Students who may only have a few more weeks experience start quickly become peer-mentors, helping new students through the initial training. We see how new students learn to engage in this safe space, making connections with other students and asking questions. Once students are trained to do geometry optimizations and molecular dynamics simulations, we divide up projects to allow students to have their own system to model. Students can make meaningful project contributions while devoting only a few hours each week. This low commitment allows students to engage while taking time-intensive introductory STEM courses. Our mantra is “opportunities, not obligations”, which helps students express and explore their interests while knowing they can shift focus when school work must take priority. We see the Nanobots Research Project as a long-term structure that can evolve to engage students in addressing various research questions, led by different PIs.

Nanobots is in its third year of implementation, and students have truly embraced this opportunity! Weekly meetings average 15 students (out of a total student body of 2200) with ∼5 upper-level students continuing from previous years. These upper-level students have formed a Nanobots Leadership Council and a formal student club as a means of ensuring that all students on campus are invited. These students have an additional weekly meeting, joined by PSU research scientists, to dive deeper into the science and plan the workshop activities for the week. Students participating in Fall 2023 were asked to report their favorite things about the Nanobots Project (Figure 4). These freeform responses can be roughly grouped into three categories to give students a sense of what they value about their experience. Students enjoy the knowledge they are gaining and the connections they make with other people. They also appreciate that they are able to handle an intimidating but important new experience. We find these responses to be promising. Student’s sense of belonging predicts their performance and retention,^74^ and this preliminary data suggests involvement in Nanobots will improve belonging.

## 4 Transforming Research Infrastructure—Getting TEM into the Hands of Undergraduates at PUIs through Low-Voltage TEM and Remote Instrument Access

As identified in the recent Perspective by PUNC,^7^ access to nanoscience instrumentation—particularly transmission electron microscopy (TEM)—is a major hurdle to advancing nanoscience research at PUIs and training undergraduates in nanoscience. It can also present a steep entry barrier to research itself. At R1 institutions that are more resource-rich, electron microscopy including STEM-EDS mapping is often routine, but typically only carried out by staff, graduate students, postdocs, and other advanced researchers. Development of new and high-quality nanomaterials, however, requires a research process where imaging directs the trajectory of a project as opposed to being a final characterization method. This approach is conceptually analogous to nuclear magnetic resonance (NMR) in organic chemistry research. The Plass lab has taken two steps to address this need that may or may not be broadly transferable, depending on funding, partnerships, and location. First, acquisition of a low-voltage, compact TEM at F&M has enabled routine access by undergraduates for research and has aided in teaching. Second, in collaboration with the Materials Characterization Laboratory at Penn State, the Plass lab helped to develop remote use of a TEM with STEM-EDS capabilities.

**Figure 5:**
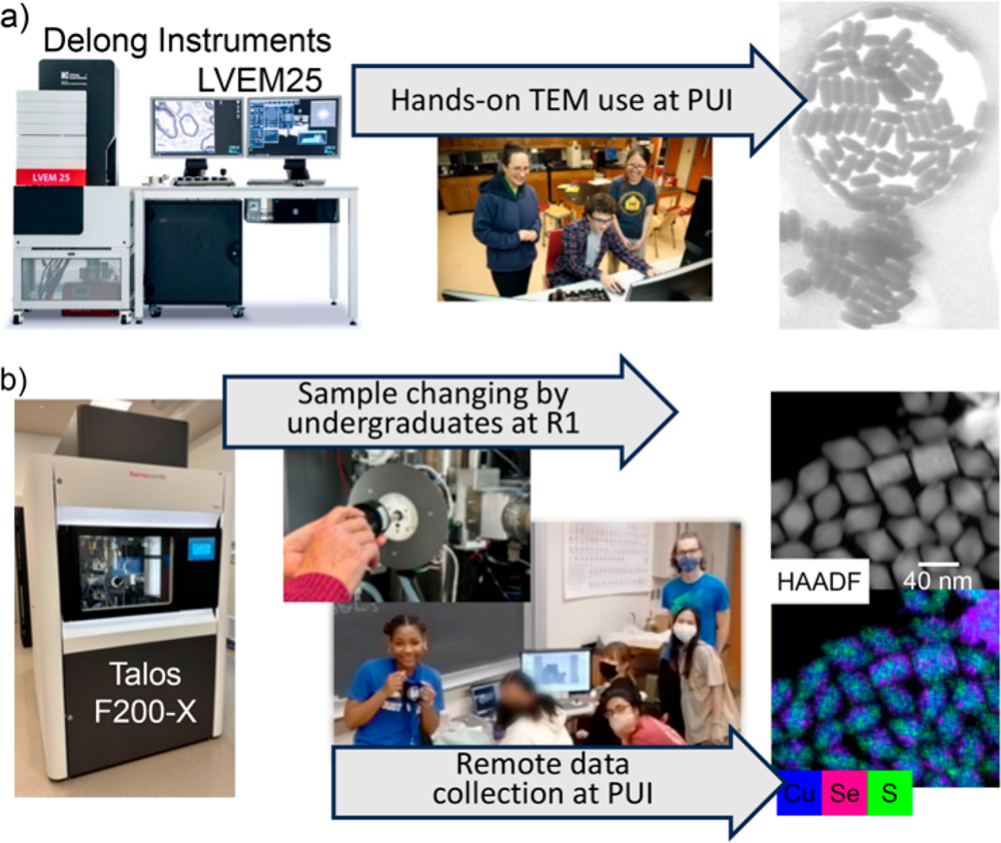
(a) Undergraduate research students at a PUI use low-voltage TEM to characterize particle size and shape (image of LVEM25 copyright Delong Instruments). Pictured left to right are Professor Katherine Plass, Angus Unruh, and Han Le (image credit Deb Grove). (b) Undergraduates at an R1 and PUI work together to do remote TEM (image of Talos F200-X copyright PSU Materials Characterization Laboratory). R1 undergraduates prepare the instrument and change samples. PUI undergraduates (shown are Alba Roselia Espinosa, Qi Rebecca Luo, Diya Dhakal, Clarisse Doligon, and Adem Imamovic; image credit Kate Plass) collect TEM, STEM, and EDS-mapping data.

Low-voltage TEM instruments allow undergraduate students routine access to this crucial nanoscience technique (Figure 5a). F&M acquired a LVEM25 microscope from Delong Instruments with the support of an NSF-MRI grant (CHE-1724948) that has supported research and class experiences for F&M and near-by PUIs.^14−16,67,75^ This is a feasible technique at many PUIs, and newer instruments with expanded SEM and EDS capabilities are available. Research students learn to use the instrument independently after two ∼1-h sessions. Obtaining size and shape measurements allows routine checks to ensure successful implementation of PST of nanoparticles rather than dissolution and regrowth, which would result in different morphologies. Given the proximity of other PUIs, students from other institutions can travel to F&M and use the instrument for classes and research.

In collaboration with the Materials Characterization Laboratory at Penn State, we have helped to develop a system for using their Thermo-Fisher Scientific Talos F200X TEM/STEM remotely, with routine data collection involving only undergraduate researchers. Undergraduates at F&M have been using this resource routinely for the past three years to do extensive STEM-EDS mapping. A microscope hand panel is located at F&M. A low-lag networking solution allows the TEM control panel at F&M to effectively operate the TEM located at Penn State, ∼100 miles away. Samples for TEM analysis are mailed to Penn State where undergraduate students that have been trained by MCL staff load samples into the TEM and provide overall support during the remote session. This arrangement bridges the necessary gap between remote access and in-person sample loading. Students at F&M learn the instrument using detailed written instructions with guidance from their peers. Figure 5b shows data collected from the F&M campus, using PSU’s STEM-EDS facilities. The ease of access to this instrument has transformed the Plass lab’s nanoscience research. We accrued 80 h of instrument time with 8 undergraduate users over the summer of 2022. Further demonstration, development, and expansion of this new capability has the potential to make advanced instrumentation more available to PUIs.

Neither of these approaches is a panacea to instrument access issues, but they do demonstrate practical pathways to making TEM routinely available to undergraduate student researchers at institutions, such as PUIs, that do not have large shared instrumentation facilities. We hope that they might inspire solutions where appropriate and perhaps motivate new creative ideas that can expand PUI instrument access more broadly. Both of these approaches require significant time and funds. Initial investments in a TEM are high, and instrumentation can require expensive repairs or service contracts. Remote TEM still requires hourly fees and frequent use to maintain training. National Science Foundation Major Research Instrumentation (MRI) and Research at Undergraduate Institution (RUI) grants have provided indispensable support for such initiatives.

## 5 Conclusion and Outlook

Despite the known challenges to undergraduate nanoscience research at PUIs, we have shown that with the innovative use of research communities, we can engage numerous undergraduates in high-impact nanomaterials research. Our focus on postsynthetic nanoparticle transformations is strategic and well suited to the model that has been developed, which combines peer–peer and near-peer mentoring with infrastructure development.

Cu_2–*x*_S nanorods are peculiarly plastic platforms that can be transformed into various shapes, compositions, and patterns, affording the potential for developing rational design principles for a variety of elaborate nanostructures. It is also a powerful platform for undergraduate-led research, providing a low entry-barrier that enables a research-team approach. It opens opportunities for computational modeling that can be merged with the creation of a fully inclusive volunteer research project that we call the nanobots Research Project. Nanobots, for us, have been an innovative approach to early career involvement of undergraduates that has the potential to be modular and broadly transferable.

A broader takeaway from this work is the virtuous cycle that can emerge from attempts to make small improvements in inclusivity. Penn State’s Materials Research Facilities Network began with the intention of enabling use of the Materials Characterization Laboratory by nearby PUI faculty at colleges located within a few hours of Penn State’s main campus. This initiated relationships that grew over years into the network of collaboration, innovation, and peer mentoring described here. Changes to the design of our experimental research group to include less experienced students had the unintended consequence of creating more flexible pathways to research that accommodated upper-level students who thought they could not do the research. The Nanobots Research Program was initiated as a way to engage first year chemistry students in research. Student engagement in the project has grown beyond the initial intention, engaging students across campus and at different levels. The computational research component is growing increasingly relevant to the scholarly work of the initiators and is integrating with their experimental work. These are hallmarks of cutting-edge nanoscience research. The ability to train students in this approach while engaging in authentic and impactful research will pay dividends in the future, from both the nanoscience research and the next generation of nanoscience researchers that it produces.
